# Supplementary material for: DREAMER-S: Deep leaRning-Enabled Attention-based Multiple-instance approaches with Explainable Representations for Spatial biology
Source: PLoS Comput Biol. 2026 May 26;22(5):e1013581. doi: 10.1371/journal.pcbi.1013581 (PMC13235922; doi:10.1371/journal.pcbi.1013581)
Supplement: S1 Table — (DOCX) [file pcbi.1013581.s001.docx]

**S1 Table.** Chemical image dataset training/testing split with 50%:50% ratio based on the ‘Patient’ column.

| SampleID | MouseID | Mouse | Replicate | Patient | Treatment | Dataset |
| --- | --- | --- | --- | --- | --- | --- |
| CRC0076_ABT_M19-1 | M19-1 | M19 | 1 | CRC0076 | ABT | Test |
| CRC0076_ABT_M19-2 | M19-2 | M19 | 2 | CRC0076 | ABT | Test |
| CRC0076_COMBO_M52-1 | M52-1 | M52 | 1 | CRC0076 | COMBO | Test |
| CRC0076_COMBO_M52-2 | M52-2 | M52 | 2 | CRC0076 | COMBO | Test |
| CRC0076_FOLFOX_M20-1 | M20-1 | M20 | 1 | CRC0076 | FOLFOX | Test |
| CRC0076_FOLFOX_M20-2 | M20-2 | M20 | 2 | CRC0076 | FOLFOX | Test |
| CRC0076_VEHICLE_M23-1 | M23-1 | M23 | 1 | CRC0076 | VEHICLE | Test |
| CRC0076_VEHICLE_M23-2 | M23-2 | M23 | 2 | CRC0076 | VEHICLE | Test |
| CRC0076_VEHICLE_M23-3 | M23-3 | M23 | 3 | CRC0076 | VEHICLE | Test |
| CRC0076_VEHICLE_M23-4 | M23-4 | M23 | 4 | CRC0076 | VEHICLE | Test |
| CRC0344_ABT_M4-1 | M4-1 | M4 | 1 | CRC0344 | ABT | Test |
| CRC0344_ABT_M4-2 | M4-2 | M4 | 2 | CRC0344 | ABT | Test |
| CRC0344_ABT_M51-1 | M51-1 | M51 | 1 | CRC0344 | ABT | Test |
| CRC0344_ABT_M51-2 | M51-2 | M51 | 2 | CRC0344 | ABT | Test |
| CRC0344_COMBO_M54-1 | M54-1 | M54 | 1 | CRC0344 | COMBO | Test |
| CRC0344_COMBO_M54-2 | M54-2 | M54 | 2 | CRC0344 | COMBO | Test |
| CRC0344_FOLFOX_M22-1 | M22-1 | M22 | 1 | CRC0344 | FOLFOX | Test |
| CRC0344_FOLFOX_M22-2 | M22-2 | M22 | 2 | CRC0344 | FOLFOX | Test |
| CRC0344_VEHICLE_M27-1 | M27-1 | M27 | 1 | CRC0344 | VEHICLE | Test |
| CRC0344_VEHICLE_M27-2 | M27-2 | M27 | 2 | CRC0344 | VEHICLE | Test |
| CRC0076_ABT_M47-1 | M47-1 | M47 | 1 | CRC0076 | ABT | Train |
| CRC0076_ABT_M47-2 | M47-2 | M47 | 2 | CRC0076 | ABT | Train |
| CRC0076_COMBO_M11-1 | M11-1 | M11 | 1 | CRC0076 | COMBO | Train |
| CRC0076_COMBO_M11-2 | M11-2 | M11 | 2 | CRC0076 | COMBO | Train |
| CRC0076_FOLFOX_M27-1 | M27-1 | M27 | 1 | CRC0076 | FOLFOX | Train |
| CRC0076_FOLFOX_M27-2 | M27-2 | M27 | 2 | CRC0076 | FOLFOX | Train |
| CRC0076_VEHICLE_M28-1 | M28-1 | M28 | 1 | CRC0076 | VEHICLE | Train |
| CRC0076_VEHICLE_M28-2 | M28-2 | M28 | 2 | CRC0076 | VEHICLE | Train |
| CRC0076_VEHICLE_M28-3 | M28-3 | M28 | 3 | CRC0076 | VEHICLE | Train |
| CRC0076_VEHICLE_M28-4 | M28-4 | M28 | 4 | CRC0076 | VEHICLE | Train |
| CRC0344_ABT_M19-1 | M19-1 | M19 | 1 | CRC0344 | ABT | Train |
| CRC0344_ABT_M19-2 | M19-2 | M19 | 2 | CRC0344 | ABT | Train |
| CRC0344_COMBO_M2-1 | M2-1 | M2 | 1 | CRC0344 | COMBO | Train |
| CRC0344_COMBO_M2-2 | M2-2 | M2 | 2 | CRC0344 | COMBO | Train |
| CRC0344_COMBO_M30-1 | M30-1 | M30 | 1 | CRC0344 | COMBO | Train |
| CRC0344_COMBO_M30-2 | M30-2 | M30 | 2 | CRC0344 | COMBO | Train |
| CRC0344_FOLFOX_M50-1 | M50-1 | M50 | 1 | CRC0344 | FOLFOX | Train |
| CRC0344_FOLFOX_M50-2 | M50-2 | M50 | 2 | CRC0344 | FOLFOX | Train |
| CRC0344_VEHICLE_M56-1 | M56-1 | M56 | 1 | CRC0344 | VEHICLE | Train |
| CRC0344_VEHICLE_M56-2 | M56-2 | M56 | 2 | CRC0344 | VEHICLE | Train |
