# Supplementary material for: DREAMER-S: Deep leaRning-Enabled Attention-based Multiple-instance approaches with Explainable Representations for Spatial biology
Source: PLoS Comput Biol. 2026 May 26;22(5):e1013581. doi: 10.1371/journal.pcbi.1013581 (PMC13235922; doi:10.1371/journal.pcbi.1013581)
Supplement: S2 Table — (DOCX) [file pcbi.1013581.s002.docx]

**S2 Table.** Wavenumber band ratios and their biological assignment, which are based on the IR peak assignments (for cellular spectra).

| Ratio | Wavenumber / Range (cm^−1^) | Biochemical  Assignment |
| --- | --- | --- |
| Ratio Amide I/II | Intensity Ratio I_1652​_ / I_1544_​ | Protein secondary structure |
| Ratio Amide I/DNA (PO_4_^−^​) | Intensity Ratio I_1652​_ / I_964_​ | Protein / DNA (PO_4_^−^​) backbone |
| Ratio Amide I/DNA (PO_2_^−^​) | Intensity Ratio I_1230​_ / I_1244_​ | Protein / DNA (PO_2_^−^​) backbone |
| Ratio Amide I/RNA (Uracil) | Intensity Ratio I_1652​_ / I_996_​ | Protein / RNA (Uracil ring) |
| Ratio Amide I/RNA (C=O) | Intensity Ratio I_1652​_ / I_1720-1745_​ | Protein / RNA (C=O stretching) |
| Ratio Amide I/Nucleic Acids | Intensity Ratio I_1652​_ / I_1230-1244_​ | Protein / Nucleic Acids (PO_2_^−^​ asymmetric stretch) |
| Ratio Amide I/Lipid (CO-O-C) | Intensity Ratio I_1652​_ / I_1070-1170_​ | Protein / Lipid (CO-O-C stretching) |
