# Supplementary material for: DREAMER-S: Deep leaRning-Enabled Attention-based Multiple-instance approaches with Explainable Representations for Spatial biology
Source: PLoS Comput Biol. 2026 May 26;22(5):e1013581. doi: 10.1371/journal.pcbi.1013581 (PMC13235922; doi:10.1371/journal.pcbi.1013581)
Supplement: S3 Table — (DOCX) [file pcbi.1013581.s003.docx]

**S3 Table.** Performance summary of validation F1 scores across all hyperparameter tuning iterations, displaying individual trial results alongside their respective mean and standard deviation (SD).

| NumBlocks | Residual | Expansion | HiddenNodes | Replicates | | | Mean ± SD |
| --- | --- | --- | --- | --- | --- | --- | --- |
|  |  |  |  | 1 | 2 | 3 |  |
| 1 | FALSE | double | 128 | 0.850 | 0.847 | 0.792 | 0.829 ± 0.033 |
| 1 | FALSE | double | 256 | 0.627 | 0.670 | 0.697 | 0.665 ± 0.035 |
| 1 | FALSE | double | 64 | 0.649 | 0.792 | 0.697 | 0.713 ± 0.073 |
| 1 | FALSE | half | 128 | 0.899 | 0.642 | 0.697 | 0.746 ± 0.135 |
| 1 | FALSE | half | 256 | 0.850 | 0.649 | 0.688 | 0.729 ± 0.106 |
| 1 | FALSE | half | 64 | 0.697 | 0.749 | 0.798 | 0.748 ± 0.051 |
| 1 | TRUE | double | 128 | 0.670 | 0.670 | 0.733 | 0.691 ± 0.036 |
| 1 | TRUE | double | 64 | 0.670 | 0.733 | 0.733 | 0.712 ± 0.036 |
| 1 | TRUE | half | 128 | 0.670 | 0.733 | 0.697 | 0.700 ± 0.032 |
| 1 | TRUE | half | 256 | 0.670 | 0.670 | 0.670 | 0.670 ± 0.000 |
| 1 | TRUE | half | 64 | 0.524 | 0.670 | 0.670 | 0.621 ± 0.085 |
| 2 | FALSE | double | 128 | 0.335 | 0.733 | 0.627 | 0.565 ± 0.206 |
| 2 | FALSE | double | 256 | 0.524 | 0.600 | 0.600 | 0.575 ± 0.044 |
| 2 | FALSE | double | 64 | 0.649 | 0.670 | 0.950 | 0.756 ± 0.168 |
| 2 | FALSE | half | 128 | 0.596 | 0.733 | 0.847 | 0.725 ± 0.125 |
| 2 | FALSE | half | 256 | 0.733 | 0.800 | 0.792 | 0.775 ± 0.036 |
| 2 | FALSE | half | 64 | 0.950 | 0.436 | 0.792 | 0.726 ± 0.263 |
| 2 | TRUE | double | 128 | 0.670 | 0.733 | 0.670 | 0.691 ± 0.036 |
| 2 | TRUE | double | 256 | 0.792 | 0.670 | 0.670 | 0.711 ± 0.070 |
| 2 | TRUE | double | 64 | 0.688 | 0.700 | 0.744 | 0.711 ± 0.030 |
| 2 | TRUE | half | 128 | 0.670 | 0.670 | 0.670 | 0.670 ± 0.000 |
| 2 | TRUE | half | 256 | 0.733 | 0.670 | 0.670 | 0.691 ± 0.036 |
| 2 | TRUE | half | 64 | 0.670 | 0.437 | 0.792 | 0.633 ± 0.180 |
| 3 | FALSE | double | 128 | 0.642 | 0.601 | 0.333 | 0.525 ± 0.168 |
| 3 | FALSE | double | 256 | 0.744 | 0.560 | 0.333 | 0.546 ± 0.206 |
| 3 | FALSE | double | 64 | 0.642 | 0.549 | 0.670 | 0.620 ± 0.064 |
| 3 | FALSE | half | 128 | 0.847 | 0.649 | 0.749 | 0.748 ± 0.099 |
| 3 | FALSE | half | 256 | 0.649 | 0.850 | 0.560 | 0.686 ± 0.148 |
| 3 | FALSE | half | 64 | 0.549 | 0.642 | 0.649 | 0.613 ± 0.056 |
| 3 | TRUE | double | 128 | 0.670 | 0.670 | 0.670 | 0.670 ± 0.000 |
| 3 | TRUE | double | 256 | 0.697 | 0.601 | 0.670 | 0.656 ± 0.049 |
| 3 | TRUE | double | 64 | 0.670 | 0.670 | 0.524 | 0.621 ± 0.085 |
| 3 | TRUE | half | 128 | 0.670 | 0.792 | 0.670 | 0.711 ± 0.070 |
| 3 | TRUE | half | 256 | 0.847 | 0.700 | 0.792 | 0.779 ± 0.074 |
| 3 | TRUE | half | 64 | 0.688 | 0.670 | 0.436 | 0.598 ± 0.141 |
